# Variability of groundwater fluoride and its proportionate risk quantification via Monte Carlo simulation in rural and urban areas of Agra district, India

**DOI:** 10.1038/s41598-023-46197-7

**Published:** 2023-11-03

**Authors:** Shahjad Ali, Manish Baboo Agarwal, Sitaram Verma, Raisul Islam, Rajesh Kumar Deolia, Shailendra Singh, Jitendra Kumar, Ali Akbar Mohammadi, Manoj Kumar Gupta, Mehdi Fattahi, P. U. Nguyen

**Affiliations:** 1 Department of Applied Sciences, Anand Engineering College, Agra, Uttar Pradesh India; 2 Department of Environmental Science and Engineering, IIT (ISM), Dhanbad, Jharkhand India; 3 Department of Civil Engineering, GLA University, Mathura, India; 4 Department of Applied Science (Mathemetics), G.L. Bajaj Group of Institutions, Mathura, India; 5 Department of Mechanical Engineering, Anand Engineering College, Agra, India; 6 Department of Mathematics & Computing, Madhav Institute of Technology & Science, Gwalior, India; 7 Department of Environmental Health Engineering, Neyshabur University of Medical Sciences, Neyshabur, Iran; 8 Department of Applied Science, Bundelkhand Institute of Engineering and Technology (BIET), Jhansi, India; 9 Institute of Research and Development, Duy Tan University, Da Nang, Vietnam; 10 School of Engineering &Technology, Duy Tan University, Da Nang, Vietnam

**Keywords:** Environmental sciences, Chemistry

## Abstract

This study quantifies the groundwater fluoride contamination and assesses associated health risks in fluoride-prone areas of the city of Taj Mahal, Agra, India. The United States Environmental Protection Agency (USEPA) risk model and Monte Carlo Simulations were employed for the assessment. Result revealed that, among various rural and urban areas Pachgain Kheda exhibited the highest average fluoride concentration (5.20 mg/L), while Bagda showed the lowest (0.33 mg/L). Similarly, K.K. Nagar recorded 4.38 mg/L, and Dayalbagh had 1.35 mg/L. Both urban and rural areas exceeded the WHO-recommended limit of 1.5 mg/L, signifying significant public health implications. Health risk assessment indicated a notably elevated probability of non-carcinogenic risk from oral groundwater fluoride exposure in the rural Baroli Ahir block. Risk simulations highlighted that children faced the highest health risks, followed by teenagers and adults. Further, Monte Carlo simulation addressed uncertainties, emphasizing escalated risks for for children and teenagers. The Hazard Quotient (HQ) values for the 5th and 95th percentile in rural areas ranged from was 0.28–5.58 for children, 0.15–2.58 for teenager, and 0.05–0.58 for adults. In urban areas, from the range was 0.53 to 5.26 for children, 0.27 to 2.41 for teenagers, and 0.1 to 0.53 for adults. Physiological and exposure variations rendered children and teenagers more susceptible. According to the mathematical model, calculations for the non-cancerous risk of drinking water (HQ-ing), the most significant parameters in all the targeted groups of rural areas were concentration (C_W)_ and Ingestion rate (IR). These findings hold relevance for policymakers and regulatory boards in understanding the actual impact and setting pre-remediation goals.

## Introduction

Fluoride is a common elements in the Earth’s crust (625 mg/kg) and, in aqueous solution, it behaves as F¯ ions^1, 2^. Elevated level of groundwater fluoride (F¯) has been reported as a major worldwide contaminant^3^. Potable groundwater serves as a significant source of exposure to fluoride for living organisms^1, 2^. According to the drinking water standard proposed by Indian Standards 10,500 (2012) and World Health Organization^4^, safe limit of fluoride in drinking water is 1.5 mg/L. However, levels exceeding this threshold are considered to be polluted^4–8^. Approximately 200 million individuals across 25 nations consume water containing elevated fluoride levels, exceeding the WHO’s tolerable limit of 1.5 mg/L^3, 6^. Fluoride contamination in groundwater can arise from natural geological formations, industrial processes, and anthropogenic activities.

The origin of fluoride contamination in groundwater can stem from either geogenic or anthropogenic sources. Geogenic sources are the most widespread cause^9–12^. Anthropogenic sources refer to activities such as combustion of coal and its by-products, bricks making industries, steel producing plants and the excessive fertilizer usage in the agriculture lands. On the other hand, geogenic sources may arise from ion exchange, rock-water interactions, rock characteristics, water vapor conversion, and calcite precipitation. Geogenic source also encompass fluoride-bearing minerals like fluorite, apatite and amphiboles, which may release fluoride into the groundwater through mechanism such as ion exchange and the rock-water interaction^9–11^. Understanding these diverse sources is crucial in formulating effective mitigation strategies and safeguarding public health.

Elevated fluoride levels (> 1.5 mg/L) can lead to severe health issues, including bone disorders, mottled dental enamel, impaired food absorption, arthritis, stress-related problems, impotence, bone cancer, skeletal fluorosis, and disorders affecting the liver, lungs, and kidneys^13–19^. Globally, groundwater fluoride contamination poses a significant challenge due to its natural occurrence. About two hundred million peoples, from 25 developing countries are severally affected by the detrimental consequences of fluorosis^16, 17, 20, 21^. In India alone, approximately 66 million individuals, includes six million children below 14 years of age, and 45 million in China, face risks such as teeth mottling, bone deformities and neurological damage by regular use of ground-drinking water containing fluoride levels ≥ 1.5 mg/L^22^. The existing body of literature provides valuable insights into the diverse sources, distribution patterns, and mitigation strategies related to groundwater fluoride contamination. Adimalla et al.^20^ conducted a study on the groundwater of Medak region in Telangana State, India. Their analysis revealed that the fluoride level in groundwater exceeded WHO’s recommended limits for safe drinking water in nearly 50% of the collected samples^20^. Sahu et al.^23^ focused their research on the Dongargaon block, Chhattisgarh, India, involving both human subjects and domestic animals. They discovered that every fifth living being in the area was suffering from fluorosis^23^. Kundu and Mandal^24^ conducted a study in the Hooghly district of West Bengal, determining that the excessive phosphate fertilizer usage led to an increase in fluoride percentage in the groundwater^24^. Pandith et al.^25^ investigated the groundwater of the Pandharkawada block in Yavatmal district, Maharashtra, India. Their analysis showed seasonal variation in groundwater fluoride levels between the pre and post-monsoon seasons across the district^25^. Egbueri^26^ conducted experiment in the northeast of Nigeria and found that the drinking water in this region is unsuitable due to its fluoride levels surpassing permissible limits. The health risk assessment revealed chronic health risk for children, females, and males due to water intake^26^. Further, Qasemi et al.^27^ conducted a survey in nine wells of Sabzevar, Iran, and performed experiments on groundwater samples. They found that more than half of the sampled area had fluoride concentrations below the permissible limit^27^. Similarly, numerous studies worldwide have reported elevated groundwater fluoride concentration such as China, Ethiopia, Ghana, India, Iran, Kenya, Mexico and Pakistan^10, 12, 16, 20, 28–34^.

Hence, the quality of groundwater is under severe threat and is of major concern. Few studies have focused on elevated level at the district level. If fluoride contamination occurs in densely populated regions, the health risk implications would be even more critical due to a higher number of potential exposures. Previous researchers have primarily conducted risk assessments using a deterministic approach. However, this study employs Monte Carlo simulations for exposure assessments of fluoride contamination in groundwater. The objective of this study is to compare the variation of groundwater fluoride levels in rural and urban areas of Agra district, Uttar Pradesh, India, and to quantify the proportional impact using the USEPA risk formulation. Additionally, this work aims to analyze the sources, distribution, and genesis of high fluoride concentrations in both rural and urban areas of Agra district. The outcomes of this investigation will be valuable in providing potential information to decision-makers for reducing the burden of prospective influx sources.

## Methodology

### Study area

**Figure 1:**
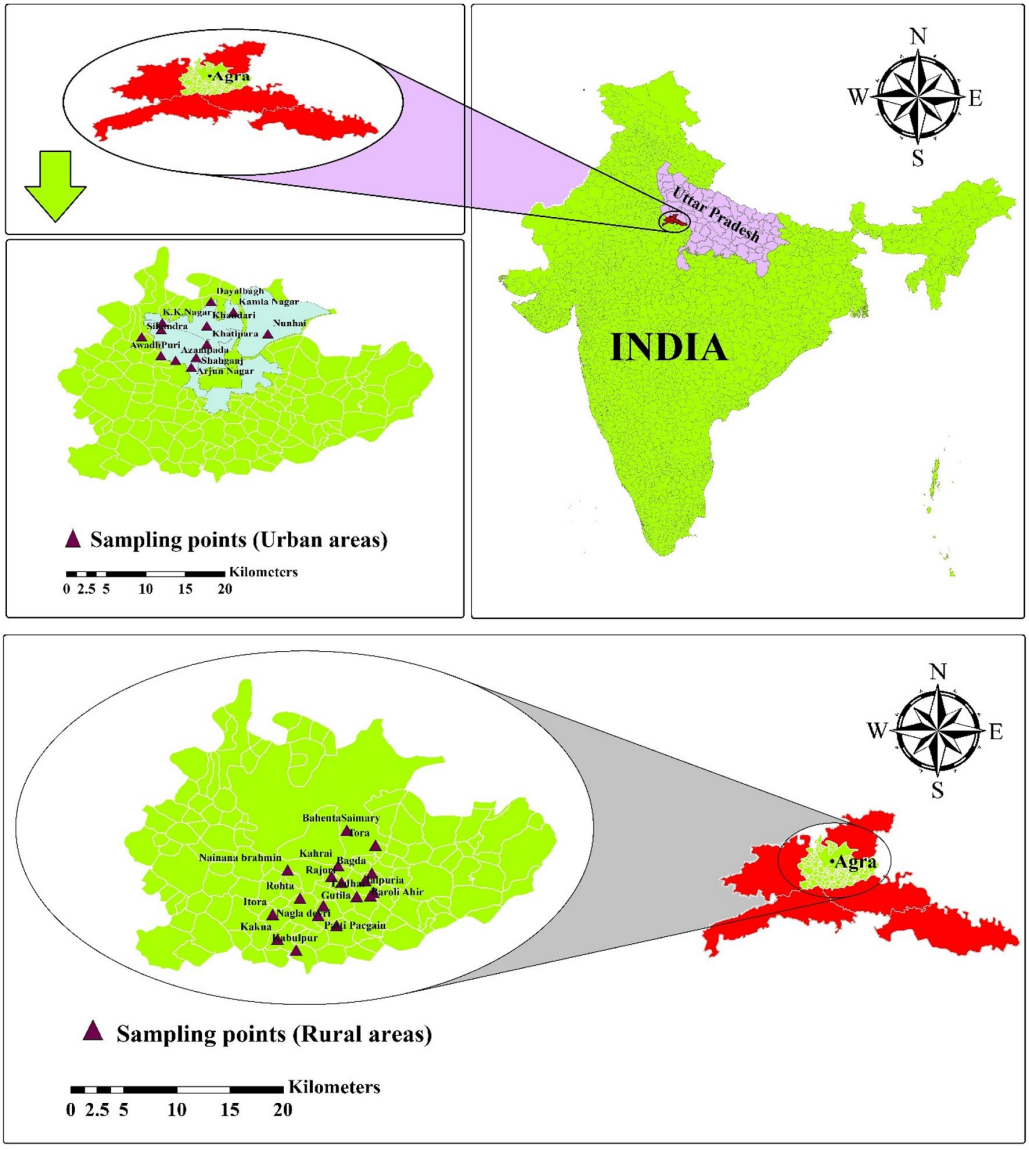
Locations of study areas (urban and rural areas) of Agra region, Uttar Pradesh, Northern India^36^.

Agra is a 23nd largest city in urban India with a large population growth of roughly 1.6 million. Agra city is situated on the banks of Yamuna river in the Northern State of Uttar Pradesh between 27°11′ N and 78°02′ E Average elevation of the area is roughly 169m above mean sea level. The climate is semi-arid to sub-tropical, with an average annual precipitation of around 687.2mm and evaporation of 1466 mm/year. The daily relative humidity varies from 30 to 100%. In the Agra region of Northern India, Baroli Ahir, representing the rural area with 170 m above sea level, and Agra city^35^, representing the urban area, were selected as the study areas (Fig. 1).

### Analysis and evaluation of sample collection

A total of 150 samples were meticulously collected, with 90 samples meticulously gathered from various sites within the Baroli Ahir region, and an additional 60 samples carefully obtained from diverse locations within Agra city. The sampling regimen spanned an entire year, commencing in January 2022 and concluding in March 2023, ensuring that seasonal variations were comprehensively captured. Figure 1 vividly portrays the meticulous distribution of the sample collection sites across the Baroli Ahir region in Agra city. Water samples were collected from all corners of the sampled area, spanning various sources including boreholes/hand pumps, tube wells, wells, puddle, ponds etc. The gathered samples from different regions within the villages were diligently stored at low temperature to ensure accurate assessment of the fluoride content in each water sample. The quantification of fluoride in theses diverse samples was performed using SPADNS (4500-F-D)^1, 36–39^.

### Calculation of exposure and risk assessment of fluoride on human health

Groundwater fluoride exposure was estimated using the USEPA 1989 model. This model, based Eqs. (1) and (2), was employed to analyze the non-carcinogenic risk posed by groundwater fluoride intake^40^. The used parameters in in estimated daily intake (EDI) calculation are detailes in Table 1.

1 $$\mathrm{EDI}=\frac{\mathrm{Cw}\times \mathrm{IR}\times \mathrm{EF}\times \mathrm{ED}}{\mathrm{BW}\times \mathrm{AT}},$$

where EDI: estimated daily intake of fluoride consumption (mg/kg/day), Cw: concentration of fluoride in potable water (mg/L), IR: ingestion rate (daily limit of consumption of water (L/d)), EF: exposure frequency (days/year), ED: exposure duration (year), BW: body weight (kg), AT: averaging time (day).

**Table 1:** The parameters values which are used in health risk assessment method^1, 41^.

Parameters	Symbol	Unit	Children (2–10)	Teenagers (11–20)	Adults (> 20)
Exposure duration	ED	Year	6	6	6
Bodyweight	BW	Kg	16	45	62
Ingestion rate	IR	L/d	1.5	2.2	2.8
Exposure frequency	EF	days/year	345	345	345
Average time	AT	days	EF*ED	EF*ED	EF*ED

The Non-carcinogenic risk due to fluoride exposure is calculated by HQ as given in Eq. (2)^2^:

2 $$\mathrm{HQ}=\frac{\mathrm{EDI}}{\mathrm{Rfd}}.$$

The reference dose (RfD) is a calculation used in risk assessment to estimate the maximum daily intake of a substance that is unlikely to result in significant adverse effects over the course of an individual’s lifetime. This value serves as a reference point for evaluating potential risks associated with exposure to that particular substance. The RfD for fluoride (0.06 mg/kg/d) was sourced from the Integrated Risk Information System’s database (USEPA, IRIS). The Hazard Quotient is determined by dividing the estimated daily intake (EDI) by the reference dosage (RfD). The HQ value provides a numerical indicator of the potential for adverse health effects. If the HQ is less than 1, it suggests that the exposure is likely to be safe. Conversely, an HQ greater than 1 indicates that the exposure may pose a risk to health, especially if sustained over an extended period^42, 43^.

### Monte Carlo simulation & sensitivity analysis

The human health risk assessment process can be assessed for variability and uncertainty in numerous parameters using Monte-Carlo simulation (MCS). Oracle Crystal Ball (version 11.1.34190) was employed to conduct 10,000 iterations of the sensitivity analysis. This technique determines exposure risk and point value by selecting the parameter values from their fitted distribution^44^. Sensitivity analysis (SA) scrutinizes variations in the output of a MCS, which may arise from fluctuations in the input data^43^. The parameters for conductinng the SA using the MCS technique are detailed in Table 2. The probability distribution functions that are used in the SA and MCS are computed by the US Environmental Protection Agency (EPA)^40^.

**Table 2:** Parameters used in MCS and uncertanty analysis of fluoride.

Parameter	Age group (years)			Probability distribution	References
	Children	Teenagers	Adults		
Ingestion rate (L/d)	1.25 ± 0.57	1.58 ± 0.69	1.95 ± 0.64	Normal	^45^
Concentration (mg/L)	Likeliest = 1.79, scale: 0.51			Min extreme	This study
Body weight (kg)	16.68 ± 1.48	46.25 ± 1.18	57.03 ± 1.10	Log normal	^15^
Exposure duration (year)	6	6	6	Fixed value	^45^
Exposure frequency (days/year)	Minimum = 185, mode = 345, maximum = 365			Triangular	^46^
Averaging time (AT)(days)	2190	2190	9125	Fixed value	^45^
Oral reference dose (RfDo) (mg/kg/day)	0.06			Fixed value	^40, 41^

## Result and discussion

### The level of fluoride contamination in Baroli Ahir block (rural area) and Agra city (urban area) of Agra region

**Figure 2:**
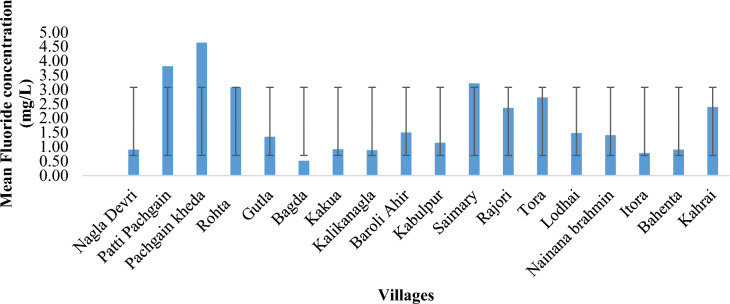
Fluoride concentration (mean) of Baroli Ahir block, Agra region, Uttar Pradesh, North India.

**Figure 3:**
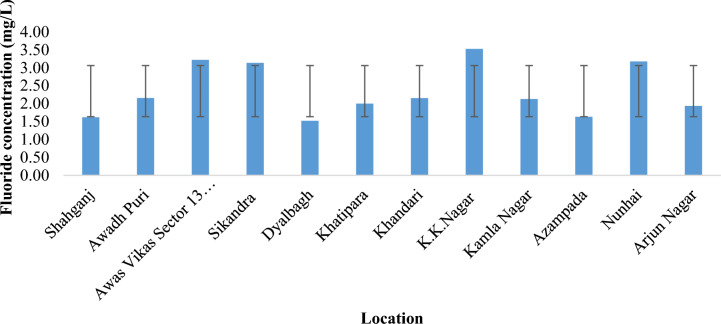
Fluoride concentration (mean) of Agra city (urban area), Agra region, Uttar Pradesh, Northern India.

Based on the analysis conducted in the sampled area, it was determined that the highest fluoride contamination in the potable water within the rural area was recorded at 5.20 mg/L in Pachgain kheda, while the lowest was 0.33 mg/L in Bagda village, averaging at 1.89 mg/L (Fig. 2). In the urban area, fluoride concentrations ranged from a maximum of 4.38 mg/L in K.K. Nagar to a minimum of 1.35 mg/L in Dayalbagh, with an average of 2.38 mg/L. Notably, this average value exceeds the WHO's acceptable limit of 1.5 mg/L (Fig. 2). Over 70% of the sampled water sources surpassed the recommended fluoride limit of 1.5 mg/L for drinking water. However, 30% of the surveyed area provided water within the acceptable limits (0.5–1.5 mg/L) for Agra district in Northern India, as depicted in Figs. 2 and 3. In a study conducted by Shahjad Ali et al. (2017) on fluoride contamination in water and its associated risk factors in rural areas of Agra district, Northern India, it was concluded that the fluoride contamination in the survey region was found to be in the limit of 0.14 to 4.88 mg/L^39^.Also, Yadav et al. in 2019 reported fluoride concentrations in groundwater of Agra city ranged between 0.90 to 4.12 mg/L with an average value of 1.88 mg/L and about 64% of water samples had concentrations beyond the permissible limit of 1.5 mg/L which might be due to geological formations and anthropogenic sources^35^.

### Evaluation of fluoride health risk on human health

#### Predestinarianism method

A mathematical tool was employed to assess the risk to human health, taking into account the pertinent influencing factors and strategies for their management^43^. This analysis was conducted to discern the potential effects of fluoride on the health of individuals in the Agra district of Northern India. Equation (2) was used to analyze the impact of contaminants on the all aged groups by calculating HQ and all the calculated data is illustrated in Tables 3 and 4.

**Table 3:** A Predestinarianism method of HQ computation was used in diverse rural areas of the Agra region.

Location	HQ		
	Children	Teenagers	Adults
Nagla Devri	1.405	0.733	0.677
Patti Pachgain	5.951	3.104	2.867
Pachgain kheda	7.247	3.780	3.492
Rohta	4.811	2.509	2.318
Gutla	2.108	1.1	1.016
Bagda	0.796	0.415	0.383
Kakua	1.421	0.741	0.684
Kalikanagla	1.374	0.717	0.662
BaroliAhir	2.343	1.222	1.129
Kabulpur	1.780	0.928	0.858
Saimary	5.014	2.615	2.416
Rajori	3.686	1.922	1.776
Tora	4.248	2.216	2.047
Lodhai	2.311	1.205	1.113
Nainana Brahmin	2.202	1.148	1.061
Itora	1.218	0.635	0.587
Bahenta	1.405	0.733	0.677
Kahrai	3.733	1.947	1.798
Mean	2.947	1.537	1.420
Standard deviation	1.857	0.969	0.895

**Table 4:** A Predestinarianism method of HQ computation was used in diverse urban areas of the Agra region.

Location	HQ		
	Children	Teenagers	Adults
Shahganj	2.530	1.32	1.219
AwadhPuri	3.373	1.76	1.625
Awas Vikas sector 13 colony	5.029	2.623	2.423
Sikandra	4.904	2.558	2.363
Dayalbagh	2.374	1.238	1.144
Khatipara	3.124	1.629	1.505
Khandari	3.358	1.751	1.618
K.K.Nagar	5.498	2.868	2.649
Kamla Nagar	3.327	1.735	1.603
Azampada	2.546	1.328	1.226
Nunhai	4.951	2.582	2.386
Arjun Nagar	3.014	1.572	1.452
Mean	3.669	1.914	1.768
Standard deviation	1.113	0.580	0.536

The hazard quotient (HQ) for fluoride was computed in terms of mg/kg/day and mg/day to assess oral exposure. In this research, HQ values were calculated for various age groups in different areas of the Agra district. The results revealed significant disparities in exposure levels among different age groups in rural areas: children (0.81–7.25), teenagers (0.42–3.78), and adults (0.38–3.49), with average concentrations of 2.95, 1.54, and 1.42 mg/L, respectively. Conversely, in urban areas, higher exposure doses were observed across all age groups: children (2.37–5.50), teenagers (1.24–2.87), and adults (1.14–2.65), with mean concentrations of 3.67, 1.91, and 1.77 mg/L, respectively. Notably, the maximum exposure dose limit was recorded in rural areas (7.25 mg/L for children), as detailed in Table 3**.**

**Figure 4:**
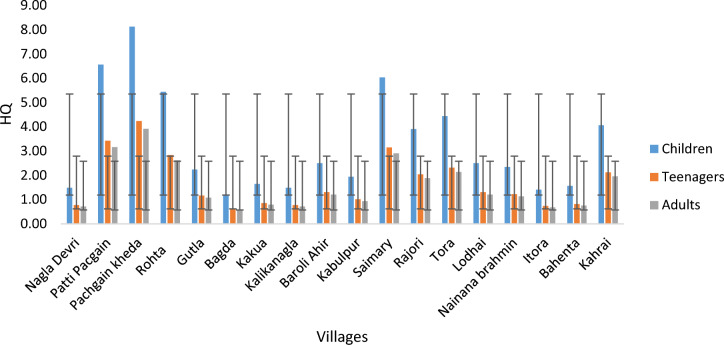
HQ values of the survey area (rural area) for the different aged groups.

**Figure 5:**
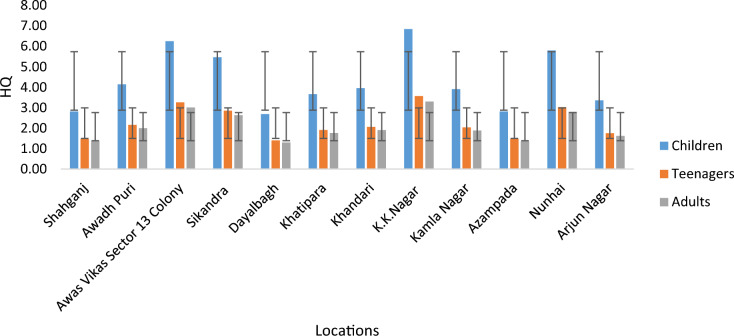
HQ values of the survey area (urban area) for the different aged groups.

However, it was observed that this range exceeded the daily fluoride limit considered ‘safe and acceptable’ by both the NRC (2001) and United States Environmental Protection Agency (USEPA) guidelines^47–50^. In accordance with USEPA recommendations, an HQ value of ≥ 1 is deemed inadvisable, as it can lead to severe non-carcinogenic health issues. Hence it is advised that the safe and clean water to be provided for the living being of that region. More than 99% of the targeted groups is having HQ value greater than 1, crossed the exceeding limit as shown in Figs. 4 and 5.

In the case study in Agra city India, Yadav et al.^35^ investigated the health risk assessment to fluoride through groundwater. Result of this study showed that the HQ value was found to be more than 1 for infants and children in all the studied areas which indicates that young consumers are more vulnerable to non-carcinogenic risk due to exposure of fluoride^35^. Results of Yousefi et al.^13^ showed that the HQ value was greater than 1 in all the studied groups of Agh Otlogh and Sari Su villages of Poldasht city, Northwest of Iran^14^.

The HQ levels of fluoride in the three exposed groups exhibited a decreasing order: children had the highest levels, followed by grown-ups, and then adults. This indicates that individuals across all age groups are experiencing hypersensitivity and other health issues as a result of consuming fluoride-contaminated water (HQ mean: 3.67) (see Table 4).

#### The probabilistic calculation by MCS methodology

**Figure 6:**
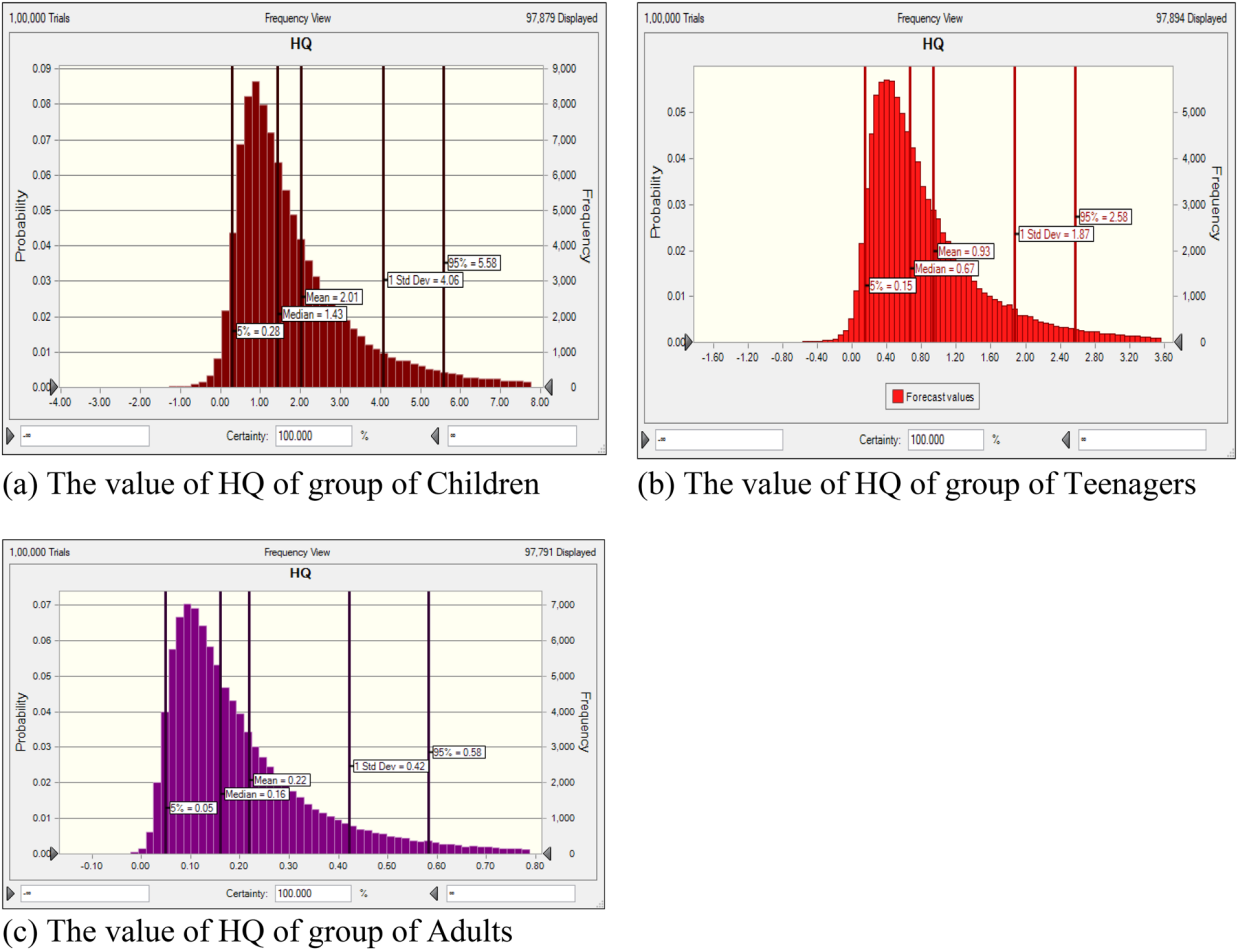
(**a–c**) Fluoride HQ’s uncertainty analysis of rural region shown by bar graphs.

**Figure7:**
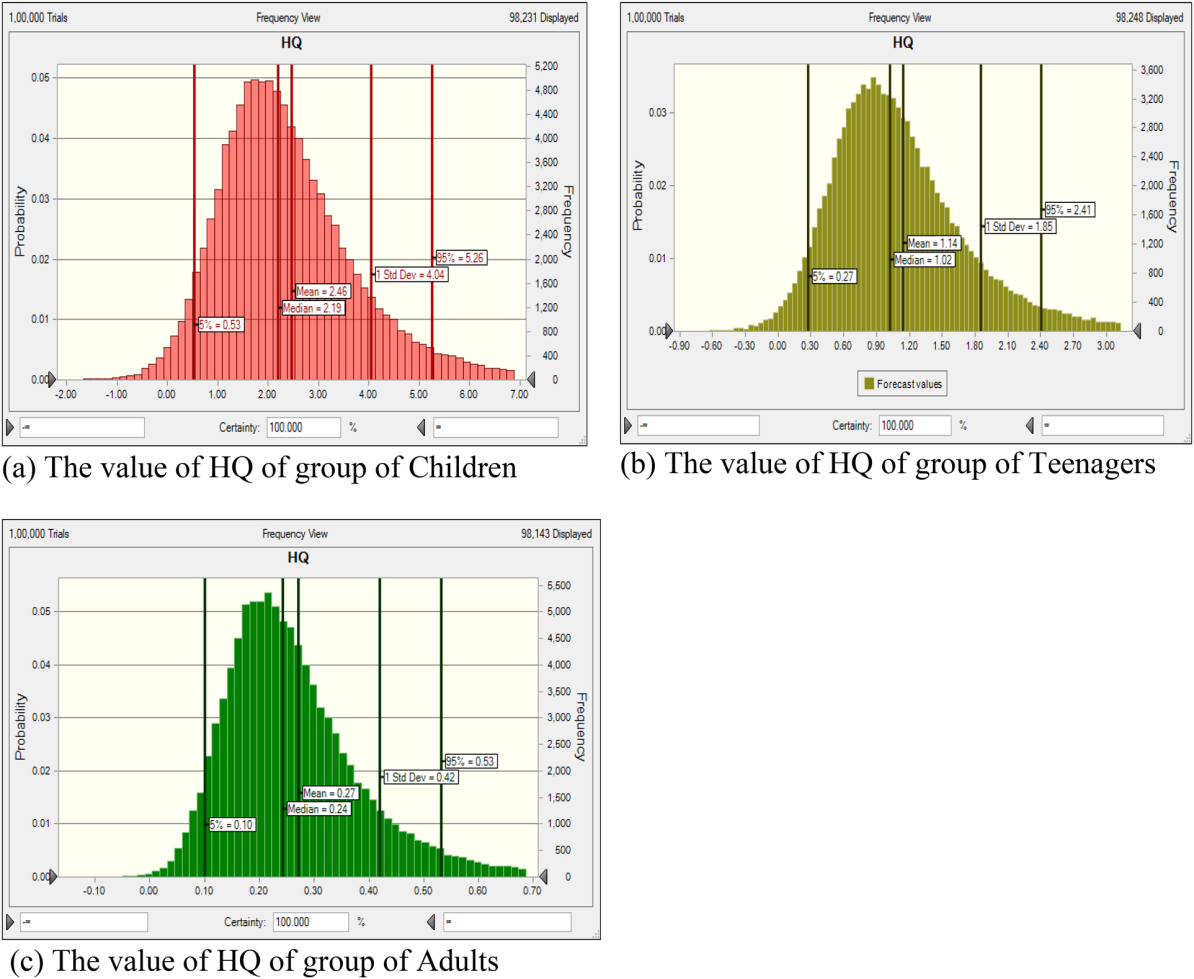
(**a–c**) Fluoride HQ’s uncertainty analysis of urban region shown by bar graphs.

HQ was determined using Eq. (2) through the MCS method. This simulation was executed using Oracle Crystal ball software (version 11.1.34190) and was run for 10,000 iterations to calculate HQ variables^43^. By appropriately considering the distribution of factors such as fluoride content, body weight (BW), ingestion rate, and exposure frequency, the MCS technique was used to assess the probabilistic approach for fluoride in all targeted groups. Figures 6a–c and 7a–c show the histograms of probability simulation for the different aged group people derived from the US EPA, for Monte Carlo simulation.

HQ values greater than 1 indicate unfavorable exposure scenarios with elevated risks of persistent non-cancer organ damage in affected individuals. The probability estimations reveal the following order of HQ values: children > teenagers > adults. As shown in Figs. 6a–c and 7a–c, the HQ for the 5th and 95th percentiles in the age groups of children, adolescents, and adults were as follows: 0.28–5.58, 0.15–2.58, and 0.05–0.58 for rural areas, and (0.53–5.26), 0.27–2.41, and 0.10–0.53 for urban areas, respectively. This indicates that children and adolescents are at an increased risk of health issues. Notably, the 95th percentile of HQ value for children was 5.58, signifying a higher level of health risk. Health risk assessment encompasses two vital components: unpredictability and sensitivity. These facets are interdependent and cannot be overlooked. Uncertainty inevitably arises from a lack of precise data concerning the various parameters under consideration. To mitigate the impact of uncertainty in health risk assessment, Monte Carlo Simulation (MCS) is employed. Given that USEPA's recommended values may vary based on geographic location or individual characteristics, ambiguity is frequently observed in risk assessment. To address this, simulations incorporate a random selection of values for each parameter. Additionally, a sensitivity analysis was conducted to gauge the extent of uncertainty, focusing on the various input factors and their potential influence on the outcome of the results^48, 49^.

**Figure 8:**
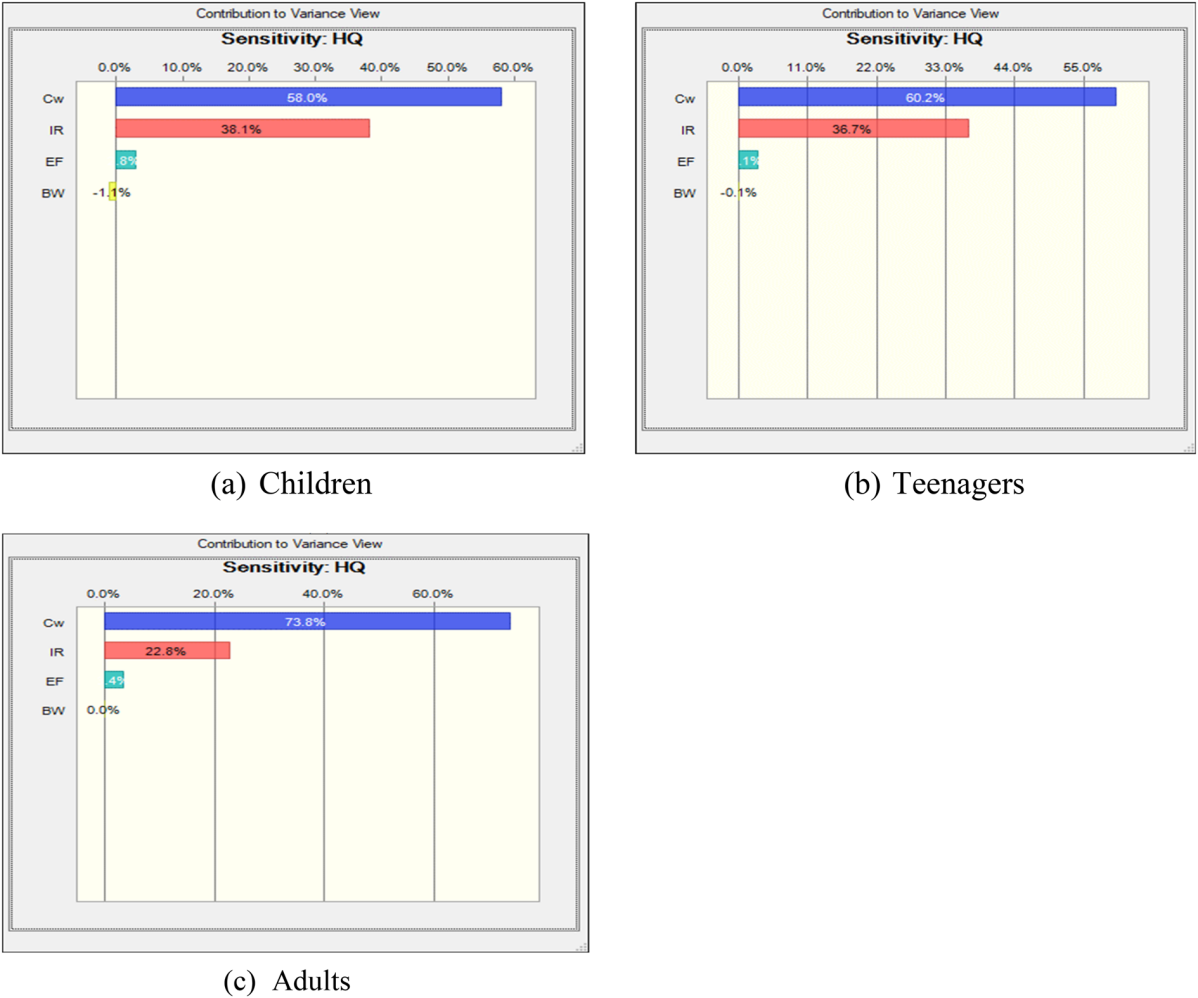
(**a**–**c**) Fluoride exposure sensitivity study of several populations (rural region).

**Figure 9:**
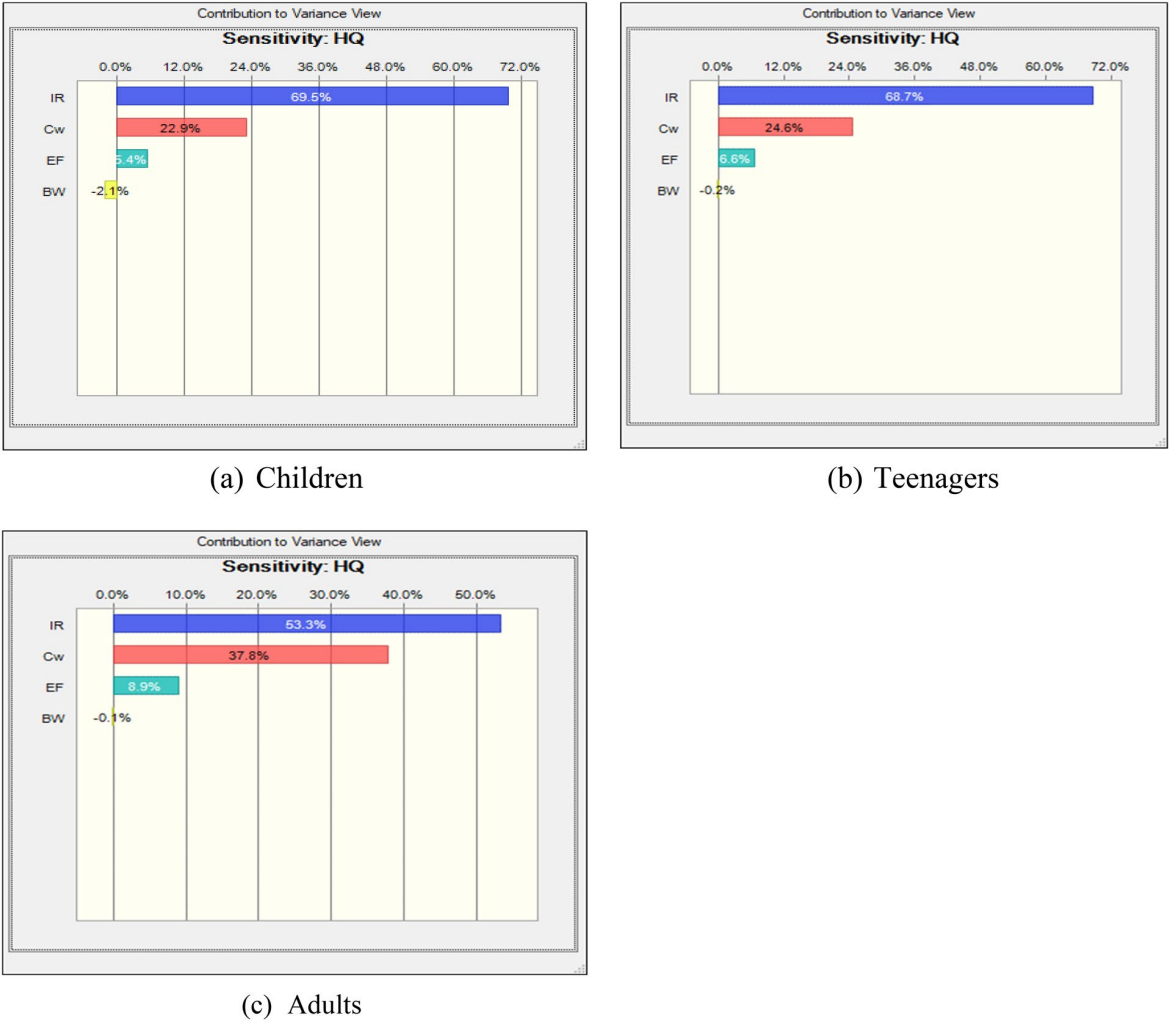
(**a**–**c**) Fluoride exposure sensitivity study of several populations (urban region).

This study aimed to assess potential health risks through a sensitivity analysis of various input parameters such as C_W_, IR, EF, AT, BW, ED, etc. These parameters were randomly selected randomly to conduct sensitivity analysis and generate tornado plots for different target groups namely children, teenagers and adults (Figs. 8a–c and 9a–c). In terms of non-carcinogenic risk through ingestion, sensitivity analysis revealed descending order of C_W_ > EF > IR > BW for children, teenagers and adults (Fig. 8a–c). However, the trend differed for urban areas, with the order of IR > C_W_ > EF > BW for different aged groups (Fig. 9a–c). A qualitative sensitivity analysis was conducted in this study to identify the most critical factors influencing the health of exposed population.

The sensitivity analysis of non-cancerous risk assessment for the targeted groups, focusing on the ingestion exposure pathway, is presented in Figs. 8a–c and 9a–c.

The sensitivity analysis of non-cancerous risk assessment for the targeted groups, focusing on ingestion exposure pathway is shown in Figs. 8a–c and 9a–c. Mathematical calculations of the non-cancerous risk of drinking water (HQ-ing) were performed using the model. In rural areas, the most influential parameters for all targeted groups were CW and IR, with correlation coefficients ranging from 58 to 73.8% and from 22.80 to 38.10%, respectively. In urban areas, the factors CW and IR exhibited ranges of influence from 22.9 to 37.8% and 53.3% to 69.5%, respectively. The probability distributions of CW and IR emerged as pivotal factors in enhancing the accuracy of the results, as highlighted in the sensitivity analysis.

## Conclusions

This study confirms the presence of F^−^ in groundwater samples collected from both rural and urban areas of Agra district. Significantly, it underscores higher concentration of groundwater fluoride in rural areas as compared to urban region within the district. Approximately, around 70% of the groundwater samples exhibited fluoride levels surpassing the permissible limit of 1.5 mg/L, potentially originating from both anthropogenic and geological origin. However, approximately 30% of the samples met the criteria for safe drinking water (0.5–1.5 mg/L).

The health risk assessment strongly indicates that oral exposure to groundwater fluoride in rural areas, particularly in the Baroli Ahir block, pose a significant threat to human health, given that groundwater serves as primary source of potable water in the study area. Rural residents face a heightened risk of fluorosis due to elevated fluoride ingestion through groundwater consumption. The estimated Hazard Quotient at 95th percentiles were notably elevated for children and teenagers in study area, signifying their increased vulnerability to health issues arising from fluoride exposure. Notably, the 95th percentile HQ value for children was 5.58, indicating a notably higher health risk in accordance with USEPA safety risk guidelines. The sensitivity analysis identified CW and IR as the predominant influential factors affecting the outcome of the results. For future research endeavors, it is recommended to include a comprehensive analysis of other sources of drinking water, as this study predominantly focused on groundwater as the primary source of drinking water in Agra city”.
